# Molecular characteristics, clonal transmission, and risk factors of *Clostridioides difficile* among hospitalized patients in a tertiary hospital in Ningbo, China

**DOI:** 10.3389/fmicb.2024.1507128

**Published:** 2024-12-02

**Authors:** Liqing Hu, Shan Lin, Meng Zhang, Mengting Cai, Yuhang Shen, Peng Zeng, Xiaojun Song, Qiao Bian, Jina Gu, Yun Luo, Yu Chen, Dazhi Jin

**Affiliations:** ^1^Department of Laboratory Medicine, The First Affiliated Hospital of Ningbo University, Ningbo, China; ^2^TEDA Institute of Biological Sciences and Biotechnology, Nankai University, Tianjin, China; ^3^School of Laboratory Medicine, Hangzhou Medical College, Hangzhou, China; ^4^Key Laboratory of Biomarkers and In Vitro Diagnosis Translation of Zhejiang Province, Hangzhou, China; ^5^Institute of Ageing Research, School of Basic Medical Sciences, Hangzhou Normal University, Hangzhou, China; ^6^Department of Clinical Laboratory, Yuedong Hospital, The Third Affiliated Hospital of Sun Yat-sen University, Meizhou, China; ^7^Laboratory Medicine Center, Department of Clinical Laboratory, Zhejiang Provincial People’s Hospital, Hangzhou Medical College, Hangzhou, China; ^8^Zhejiang Provincial Center for Disease Control and Prevention, Hangzhou, China; ^9^Department of Infectious Diseases, Ningbo No.2 Hospital, Ningbo, China; ^10^School of Biotechnology and Biomolecular Sciences, University of New South Wales, Sydney, NSW, Australia

**Keywords:** *Clostridioides difficile* infection, molecular characteristics, whole-genome sequencing, clonal transmission, risk factors

## Abstract

**Background:**

Nosocomial transmission of *Clostridioides difficile* infection (CDI) has been documented in Ningbo, China. However, data on molecular characteristics, clonal transmission, and risk factors of CDI in this region remain limited.

**Methods:**

A cross-sectional study enrolled hospitalized patients with diarrhea during September to November 2021. Stool samples from all patients were tested for *C. difficile*, and isolated strains underwent toxin gene identification, genotyping, and antibiotic susceptibility testing. Whole-genome sequencing and epidemiological variables were analyzed in patients infected with *C. difficile* strains of the same sequence types (STs) to identify nosocomial transmission and risk factors for CDI.

**Results:**

Of the 907 investigated patients, 115 (12.7%) had microbiologically proven CDI, as their diarrhea was associated with toxigenic *C. difficile* strains, which comprised 106 A^+^B^+^CDT^−^, 3 A^−^B^+^CDT^−^, and 6 A^+^B^+^CDT^+^. Predominant genotypes (ST2, ST3, ST35, and ST54) exhibited distinct antibiotic resistance patterns. ST54 strains showed higher resistance to erythromycin (100%) but lower resistance to moxifloxacin (18.2%) and gatifloxacin (18.2%) (*χ*^2^ = 10.24–16.65, *p* < 0.05). ST35 strains exhibited higher resistance to ciprofloxacin (66.7%) and tetracycline (33.3%) than other STs (*χ*^2^ = 13.30–20.19, *p* < 0.05). Genomic and epidemiological analysis revealed two nosocomial clonal transmission events caused by 5 ST35 strains (with ≤2 single nucleotide polymorphism differences), elucidating clonal transmission among different floors and buildings within the hospital. Prolonged hospitalization (> 10 days) (odds ratio [95% confidence interval], 1.76 [1.05–2.93]) and penicillin-class antibiotics (1.69 [1.11–2.58]) were risk factors for CDI, with the latter being an independent risk factor (1.57 [1.02–2.42]). For *C. difficile* ST35 infection, intensive care unit (12.00 [2.77–52.05]) and neurology departments (8.08 [1.46–44.65]) admissions were risk factors, with the latter as an independent risk factor (1.56 [1.01–2.40]).

**Conclusion:**

Multiple *C. difficile* genotypes with varied antibiotic resistance patterns circulated in Ningbo, with ST35 causing nosocomial clonal transmission among different floors and buildings within the hospital. These findings and the identified risk factors necessitate enhanced surveillance and infection control in the region.

## 1 Introduction

*Clostridioides difficile*, a major nosocomial pathogen causing antibiotic-associated diarrhea, produces highly resistant spores that persist in hospital environments (Bi et al., 2023). *C. difficile* infection (CDI) manifests clinically from mild diarrhea to pseudomembranous colitis and even death (Shu et al., 2023). Posing an urgent threat to the U.S. healthcare system and public health, CDI resulted in approximately 223,900 cases, 12,800 deaths, and US$ 1 billion in medical costs in 2019 (Wu et al., 2022). Therefore, continuous surveillance of CDI and monitoring of its molecular epidemiology is crucial to detection of outbreaks and intuition of mitigation efforts toward its associated public health burden.

CDI has been an escalating concern in China, with nosocomial infection rates exceeding 10% (Wen et al., 2023). Despite variations in genotypes and antibiotic resistance among Asian countries (Luo et al., 2019), data on molecular characteristics of CDI across China still remain limited. Whole-genome sequencing (WGS) has revealed several nosocomial transmissions of specific STs in China. Previous studies have identified nosocomial transmission of ST1 in Beijing (Jia et al., 2016), ST81 in Shanghai (Qin et al., 2017), ST35 and ST37 in Zhejiang (Bi et al., 2023; Luo et al., 2024), suggesting both localized distribution and widespread dissemination of CDI. Common risk factors for CDI include advanced age, inappropriate antibiotic use, and proton pump inhibitor (PPI) administration (McDonald et al., 2018). In China, additional unique risk factors have been identified, such as lower age thresholds (Jin et al., 2017) and chronic diseases (Dai et al., 2020), and comorbidities (Saldanha et al., 2020). However, the risk factors of patients infected with nosocomial transmission-associated genotypes remain to be elucidated.

Ningbo, a major southeastern port city, has reported nosocomial transmission of multiple *C. difficile* genotypes, including ST3 and ST54 (Shu et al., 2023). To comprehensively understand the regional epidemiology of CDI, a cross-sectional study was conducted in a tertiary hospital. CDI prevalence, molecular characteristics, and clonal transmission were studied through genomic and epidemiological data. Furthermore, risk factors for CDI and for genotypes associated with nosocomial transmission were investigated to inform possible strategies for preventing and controlling CDI outbreaks.

## 2 Materials and methods

### 2.1 Study design and collection of stool samples

From September to November 2021, hospitalized patients with diarrhea were enrolled in this cross-sectional study at the Ningbo First Hospital, an academic tertiary care medical center. Diarrhea was defined as more than 3 loose, watery, or unformed stool passages within 24 h (McDonald et al., 2018). Stool samples were collected from hospitalized patients presenting with diarrhea. Only the first stool sample from each patient was included, exclusively for the purpose of research testing.

A case of CDI was defined as the presence of diarrhea concomitant with either a positive stool assay for toxigenic *C. difficile* or endoscopic/histopathological evidence of pseudomembranous colitis (McDonald et al., 2018). Parallel to stool collection, a standardized questionnaire was completed by clinicians for each patient with CDI to record clinical data (e.g., age, gender, previous antibiotic treatment within 8 weeks, hospital stay before sampling, and past medical history) as previously described (Jin et al., 2017). Ethical approval for this study was obtained from the Ethics Committee of Ningbo First Hospital (2021-R177).

### 2.2 *C. difficile* culture

Stool samples were treated with 95% alcohol, and the mixture was inoculated on cycloserine-cefoxitin-fructose agar with a selective supplement (Oxoid Inc., Basingstoke, UK), as previously described (Jin et al., 2017). After incubation for 48 h at 37°C in an anaerobic chamber (DW Microbiology Co., Ltd., Hangzhou, China), *C. difficile* strains were identified as described previously and stored at −80°C in brain-heart infusion broth with 10% glycerol until subsequent analysis (McDonald et al., 2005).

### 2.3 Detection of *C. difficile* toxin genes

Genomic DNA was extracted using a QIAamp DNA Mini Kit (Qiagen Inc., CA, USA). The housekeeping gene *tpi*, toxin genes *tcdA* and *tcdB*, and binary toxin genes (CDT) *cdtA* and *cdtB* were detected by PCR using previously described primer sequences (Persson et al., 2008). For the *tcdA* primers, a 369-bp amplicon was obtained for the *tcdA*-positive/*tcdB*-positive (A^+^B^+^) strains, and a 100-bp amplicon was obtained for the *tcdA-*negative/*tcdB*-positive (A^−^B^+^) strains. Standard *C. difficile* strains (ATCC 43255, ATCC 700057, BAA-1801, BAA-1803, and BAA-1870) were used as positive and negative controls (Jin et al., 2017). Blank, positive, and negative controls were examined in parallel for each test.

### 2.4 Multi-locus sequence typing (MLST)

Seven housekeeping genes (*adk*, *atpA*, *dxr*, *glyA*, *recA*, *sodA*, and *tpi*) were amplified by PCR and MLST was performed as previously reported (Griffiths et al., 2010). The sequencing results were submitted to the MLST database^1^ to obtain the *C. difficile* alleles and STs. Based on the STs, the minimum spanning tree was constructed using the MSTree V2 algorithm with the GrapeTree software (Fernandes et al., 2018).

### 2.5 WGS and assembly

Genomic DNA was extracted from strains with identical STs collected from the same ward, using the method described previously. WGS libraries were prepared using the TruePrep™ DNA library prep kit V2 (Illumina, Santiago, CA, USA), and WGS was performed by the Illumina Hiseq X Ten platform with 150-base paired end reads. Sequence data were processed and quality controlled according to a standard pipeline as previously described (Preston et al., 2014). Raw sequence data were processed and quality controlled, as previously described (Bolger et al., 2014). Briefly, FASTQ-formatted Illumina sequence reads were quality controlled with a minimum quality Phred score of 30 (as a rolling average over four bases), and adapters and low-quality sequences were removed using Trimmomatic v0.36, as previously described (Bolger et al., 2014). Totally, 96 Gb of clean bases were ultimately generated (1.32 Gb/per isolate, Q20 ≥ 95%). Genomic sequence data were *de novo* assembled using Velvet 1.2.10. Raw data were deposited in the NCBI database under the BioProject accession number PRJNA902108.

### 2.6 SNP calling and identification of clonal transmission

The genome sequences of all sequenced strains were aligned to the *C. difficile* publicly available completed genomes as described in the “Results” section. Single nucleotide polymorphism (SNP) identification was performed using a section of the recombination event tree (SaRTree) pipeline at a proportion threshold of 100 (Hu et al., 2020). In accordance with the guiding principles, pairs of strains that differed by 0–2 SNPs and were separated by less than 124 days were considered the result of direct clonal transmission, whereas pairs of strains that differed by 3–10 SNPs were considered potential clonal transmission (Eyre et al., 2013).

### 2.7 Antibiotic susceptibility testing

Antibiotic susceptibility testing was performed using the agar dilution assay according to the Clinical and Laboratory Standards Institute (CLSI) guidelines (CLSI, 2020). The 12 tested antibiotics were clindamycin, ciprofloxacin, erythromycin, fusidic acid, gatifloxacin, levofloxacin, metronidazole, moxifloxacin, piperacillin, rifampicin, tetracycline, and vancomycin, with *C. difficile* ATCC 700057 and *Bacteroides fragilis* ATCC 25285 included as control strains. Minimal inhibitory concentration (MIC) results were interpreted using the CLSI M100 recommendations for clindamycin, metronidazole, moxifloxacin, piperacillin, and tetracycline (CLSI, 2020); EUCAST guidelines^2^ for fusidic acid, rifampicin, and vancomycin; and CLSI recommendations for moxifloxacin in anaerobes applied to ciprofloxacin, gatifloxacin, and levofloxacin. The erythromycin breakpoint was set at 8 μg/mL as previously reported (Mutlu et al., 2007). Multidrug resistance (MDR) was defined as resistance to at least three antibiotic classes (Magiorakos et al., 2012).

### 2.8 Data analysis

Statistical analysis was conducted using SPSS Statistics 26.0 (IBM Corp., NY, USA). The *χ*^2^ test or Fisher’s exact test was employed to assess correlations between STs and antibiotic susceptibility patterns. Bivariate analysis used the *χ*^2^ test or Fisher’s exact test to evaluate differences between CDI and non-CDI cases, and Fisher’s exact test to evaluate differences between nosocomial transmission STs and non-nosocomial transmission STs. For these comparisons, odds ratios (ORs), 95% confidence intervals (CIs), and *p*-values were calculated. Variables showing significance in bivariate analysis were further examined using bivariate logistic regression to identify independent risk factors for CDI. A *p*-value <0.05 was considered statistically significant.

## 3 Results

### 3.1 Molecular characteristic

#### 3.1.1 Collection and genotyping of *C. difficile* strains

**Figure 1:**
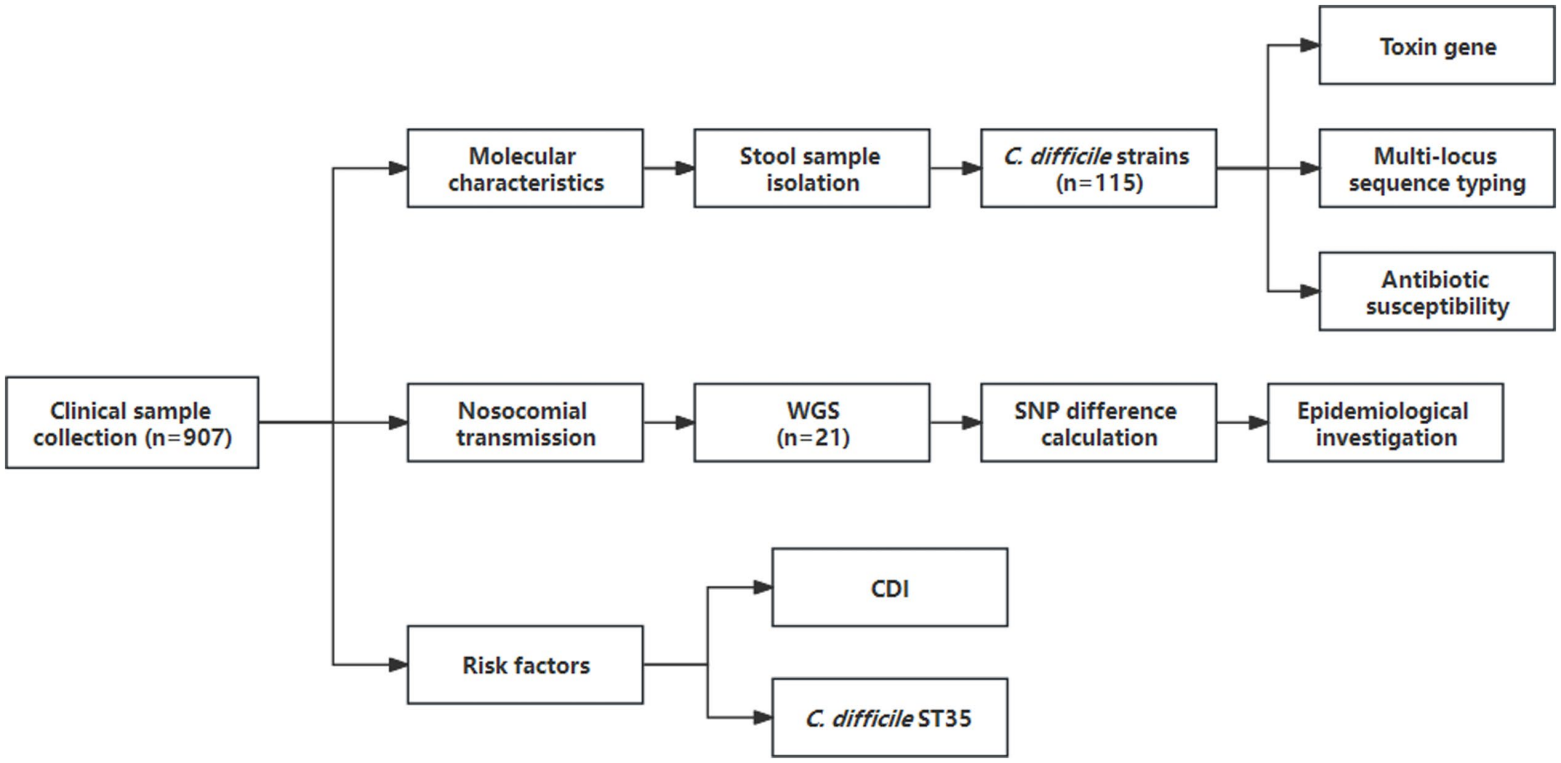
Flow diagram of data collected during the cross-sectional study (1 September 2021 to 30 November 2021). WGS, whole-genome sequencing; SNP, single nucleotide polymorphism.

A total of 907 investigated patients with diarrhea from the Ningbo First Hospital were enrolled in this cross-sectional study. The flow diagram and clinical information were shown in Figure 1 and Supplementary Table S1, respectively. Among them, 115 *C. difficile* strains were recovered with a prevalence of 12.7%. Toxin gene analysis revealed that 106 (92.2%) strains were A^+^B^+^CDT^−^, 3 (2.6%) were A^−^B^+^CDT^−^, and 6 (5.2%) were A^+^B^+^CDT^+^, and non-toxigenic strains were not found. MLST analysis identified 25 different STs, with ST3 (*n* = 16, 13.9%), ST35 (*n* = 15, 13.0%), and ST2 (*n* = 12, 10.4%) being the predominant genotypes.

#### 3.1.2 Antibiotic susceptibility testing

**Figure 2:**
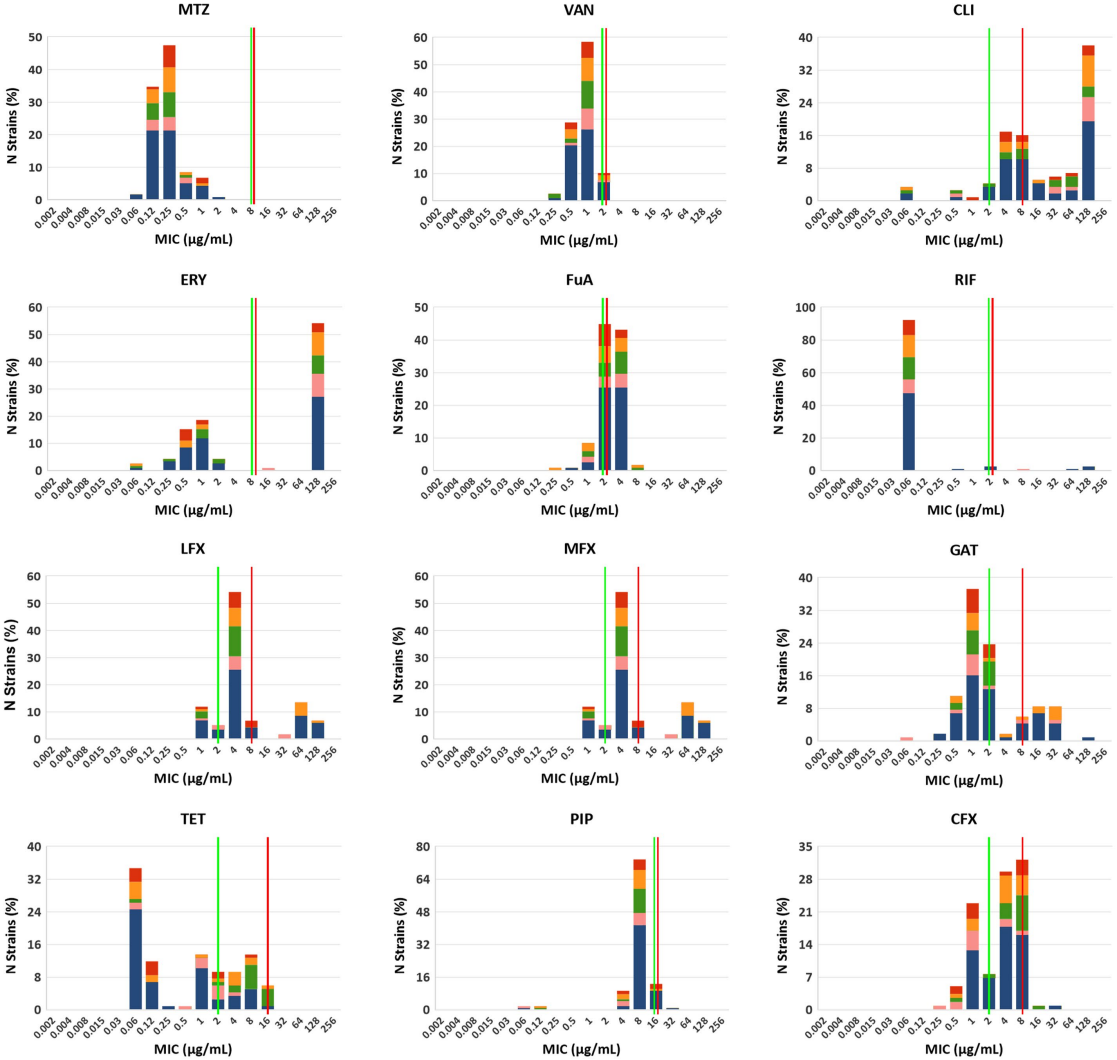
MIC distributions for 12 antibiotics among different STs, red: ST2, orange: ST3, green: ST35, pink: ST54 and blue: other STs. MTZ, metronidazole; VAN, vancomycin; CLI, clindamycin; ERY, erythromycin; FuA, fusidic acid; RIF, rifampicin; LFX, levofloxacin; MFX, moxifloxacin; GAT, gatifloxacin; TET, tetracycline; PIP, piperacillin; CFX, ciprofloxacin. Established susceptible and resistant breakpoints were indicated by vertical green and red lines, respectively, where available.

The summary MIC distributions of the 115 *C. difficile* strains for the 12 antibiotics were presented in Figure 2, and the values of the MIC_50_, MIC_90_, MIC mode and geometric mean MIC (GM) were shown in Table 1. ST35 had higher GM values for ciprofloxacin (6.35 μg/mL) and tetracycline (6.33 μg/mL) compared to all the other STs (2.84 μg/mL, 0.43 μg/mL, respectively). The ST54 had higher GM values for clindamycin (56.42 μg/mL) and erythromycin (105.95 μg/mL) compared to all the other STs (18.61 μg/mL, 12.54 μg/mL, respectively). The GM values of ST1 against metronidazole, vancomycin, rifampicin, piperacillin, and three quinolones (levofloxacin, moxifloxacin, and gatifloxacin) were higher than those of other STs.

**Table 1:** MIC parameters of genotypes against 12 antibiotic agents.

Antibiotic agent*^a^*	MIC value (μg/mL)	Total no. of strains (*n* = 115)	Genotypes					
			ST1 (*n* = 3)	ST2 (*n* = 12)	ST3 (*n* = 16)	ST35 (*n* = 15)	ST54 (*n* = 11)	Other STs (*n* = 58)
Metronidazole	MIC_range_	0.06–2	0.25–2	0.06–1	0.125–1	0.125–0.5	0.125–0.5	0.06–1
	MIC_50_	0.25	—	0.25	0.25	0.25	0.25	0.25
	MIC_90_	0.5	—	1	0.5	0.25	0.5	0.5
	MIC_mode_	0.25	0.25	0.25	0.25	0.25	0.25	0.125
	GM	0.23	0.50	0.26	0.23	0.20	0.22	0.22
Vancomycin	MIC_range_	0.25–2	1–2	0.5–2	0.5–2	0.25–1	0.5–2	0.25–2
	MIC_50_	1	—	1	1	1	1	1
	MIC_90_	2	—	1	2	1	1	2
	MIC_mode_	1	2	1	1	1	1	1
	GM	0.85	1.59	0.84	0.92	0.76	1.00	0.82
Clindamycin	MIC_range_	0.06–128	8–128	1–128	0.06–128	0.06–128	0.5–128	0.06–128
	MIC_50_	32	—	8	128	8	128	16
	MIC_90_	128	—	128	128	128	128	128
	MIC_mode_	128	—	4	128	8	128	128
	GM	18.91	25.40	14.25	25.70	10.53	56.42	17.17
Erythromycin	MIC_range_	0.06–128	1–128	0.5–128	0.06–128	0.06–128	16–128	0.06–128
	MIC_50_	128	—	1	128	2	128	2
	MIC_90_	128	—	128	128	128	128	128
	MIC_mode_	128	128	0.5	128	128	128	128
	GM	11.40	25.40	5.66	15.28	7.98	105.95	8.39
Fusidic acid	MIC_range_	0.25–8	2–4	2–4	0.25–8	1–8	1–4	0.5–4
	MIC_50_	2	—	2	2	4	2	2
	MIC_90_	4	—	4	4	4	4	4
	MIC_mode_	2	—	2	2	4	4	4
	GM	2.53	2.52	2.38	2.09	2.89	2.30	2.63
Rifampicin	MIC_range_	0.06–128	0.06–128	0.06–0.06	0.06–0.06	0.06–0.06	0.06–8	0.06–128
	MIC_50_	0.06	—	0.06	0.06	0.06	0.06	0.06
	MIC_90_	0.06	—	0.06	0.06	0.06	0.06	2
	MIC_mode_	0.06	0.06	0.06	0.06	0.06	0.06	0.06
	GM	0.09	0.77	0.06	0.06	0.06	0.09	0.11
Tetracycline	MIC_range_	0.06–16	0.06–0.125	0.06–8	0.06–16	0.06–16	0.06–4	0.06–16
	MIC_50_	1	—	0.125	1	8	1	0.125
	MIC_90_	8	—	2	8	16	2	8
	MIC_mode_	0.06	0.06	0.06	0.06	8	2	0.06
	GM	0.53	0.08	0.21	0.73	6.33	0.82	0.32
Piperacillin	MIC _range_	0.06–32	8–16	4–16	0.125–16	0.125–8	0.06–8	0.06–32
	MIC_50_	8	—	8	8	8	8	8
	MIC_90_	16	—	16	8	8	8	16
	MIC_mode_	8	16	8	8	8	8	8
	GM	7.09	12.70	8.48	5.66	6.06	4.24	8.09
Levofloxacin	MIC_range_	1–128	4–128	1–8	1–128	1–4	1–32	1–128
	MIC_50_	4	—	4	4	4	4	4
	MIC_90_	64	—	8	64	4	32	64
	MIC_mode_	4	128	4	4	4	4	4
	GM	6.64	40.32	4.00	12.88	3.03	4.54	7.36
Moxifloxacin	MIC_range_	0.125–128	1–32	0.125–2	1–32	1–2	0.5–16	0.125–128
	MIC_50_	1	—	1	1	1	1	1
	MIC_90_	16	—	2	32	2	8	16
	MIC_mode_	1	—	1	1	1	1	1
	GM	1.98	8.00	0.84	4.18	1.15	1.37	2.20
Gatifloxacin	MIC_range_	0.06–128	1–32	0.25–2	0.5–32	0.5–2	0.06–32	0.25–128
	MIC_50_	1	—	1	2	1	1	2
	MIC_90_	16	—	2	32	2	8	16
	MIC_mode_	1	32	1	1	1	1	1
	GM	2.18	10.08	1.12	4.00	1.32	1.28	2.45
Ciprofloxacin	MIC_range_	0.25–32	2–8	0.5–8	0.5–8	2–16	0.25–8	1–32
	MIC_50_	4	—	1	4	8	1	4
	MIC_90_	8	—	8	8	8	4	8
	MIC_mode_	8	—	1	4	8	1	4
	GM	3.20	4.00	2.24	3.36	6.35	1.21	3.38

—, data not calculated.

Correlations between antibiotic resistance and predominant STs were also determined (Supplementary Table S2). For ST54, the rate of erythromycin resistance (100%) was significantly higher than those in other STs (*χ*^2^ = 10.24, *p* = 0.02). However, the rates of resistance to moxifloxacin (18.2%) and gatifloxacin (18.2%) were significantly lower than those in other STs (*χ*^2^ = 16.65 and 14.91, *p* = 0.001 and 0.002, respectively). For ST35, the rates of ciprofloxacin resistance (66.7%) and tetracycline resistance (33.3%) were distinctly higher than those in other STs (*χ*^2^ = 13.30 and 20.19, *p* = 0.004 and < 0.001, respectively). Notably, of 7 tetracycline-resistant strains, 5 belonged to ST35. Moreover, 80 (69.6%) of strains were MDR with significant differences in the distribution of STs (*χ*^2^ = 9.88, *p* = 0.02).

### 3.2 Clonal transmission

#### 3.2.1 Genetic relatedness of STs across different departments

**Figure 3:**
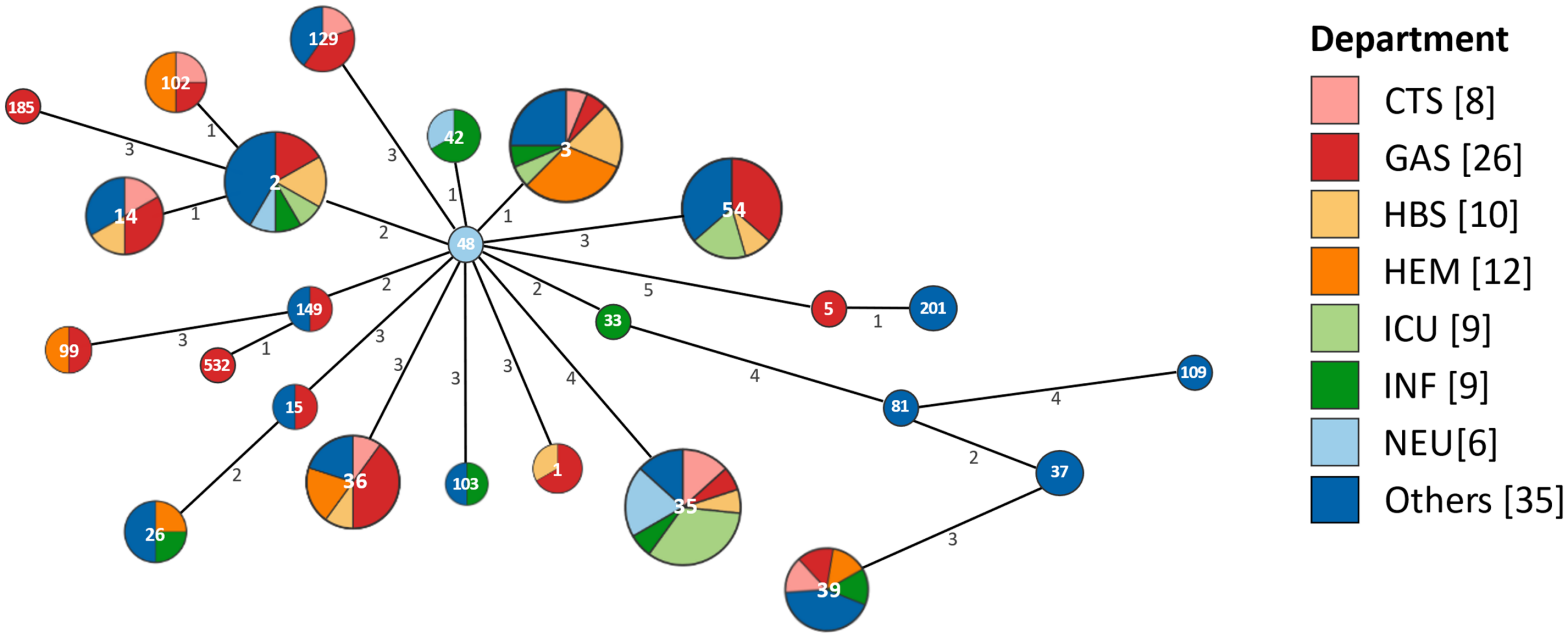
The minimum spanning tree based on the distribution of STs across different departments. Each circle represents an ST, with the size of the circle and the number of its segments indicating the quantity of corresponding isolates. The numerical values on the lines connecting the circles represent the number of allelic differences between adjacent STs. CTS, cardiothoracic surgery department; GAS, gastroenterology department; HBS, hepatobiliary surgery department; HEM, hematology department; ICU, intensive care unit; INF, infectious diseases department; NEU, neurology department.

The minimum spanning tree revealed the same STs distributed across multiple departments (Figure 3). Among these, ST48 detected in the neurology department (NEU), was identified as a putative ancestor of all STs. Further analysis showed that ST48 exhibited one allelic difference from both ST42 and ST3. Specifically, ST42 was detected in the infectious disease department (INF) and NEU, while ST3 was detected in INF, cardiothoracic surgery (CTS), hepatobiliary surgery (HBS), intensive care unit (ICU), hematology (HEM), gastroenterology (GAS), and other departments. The distribution of the main hospitalized departments was illustrated in Supplementary Figure S1. Building A mainly contained ICU, HBS, GAS, and NEU, while Building B mainly contained ICU, CTS, and HEM. A connecting platform linked Building A and Building B. The two INF were situated in Buildings C and D. The other departments were distributed across these four buildings.

#### 3.2.2 Analysis of genetic relationship and identification of clonal transmission of CDI

**Figure 4:**
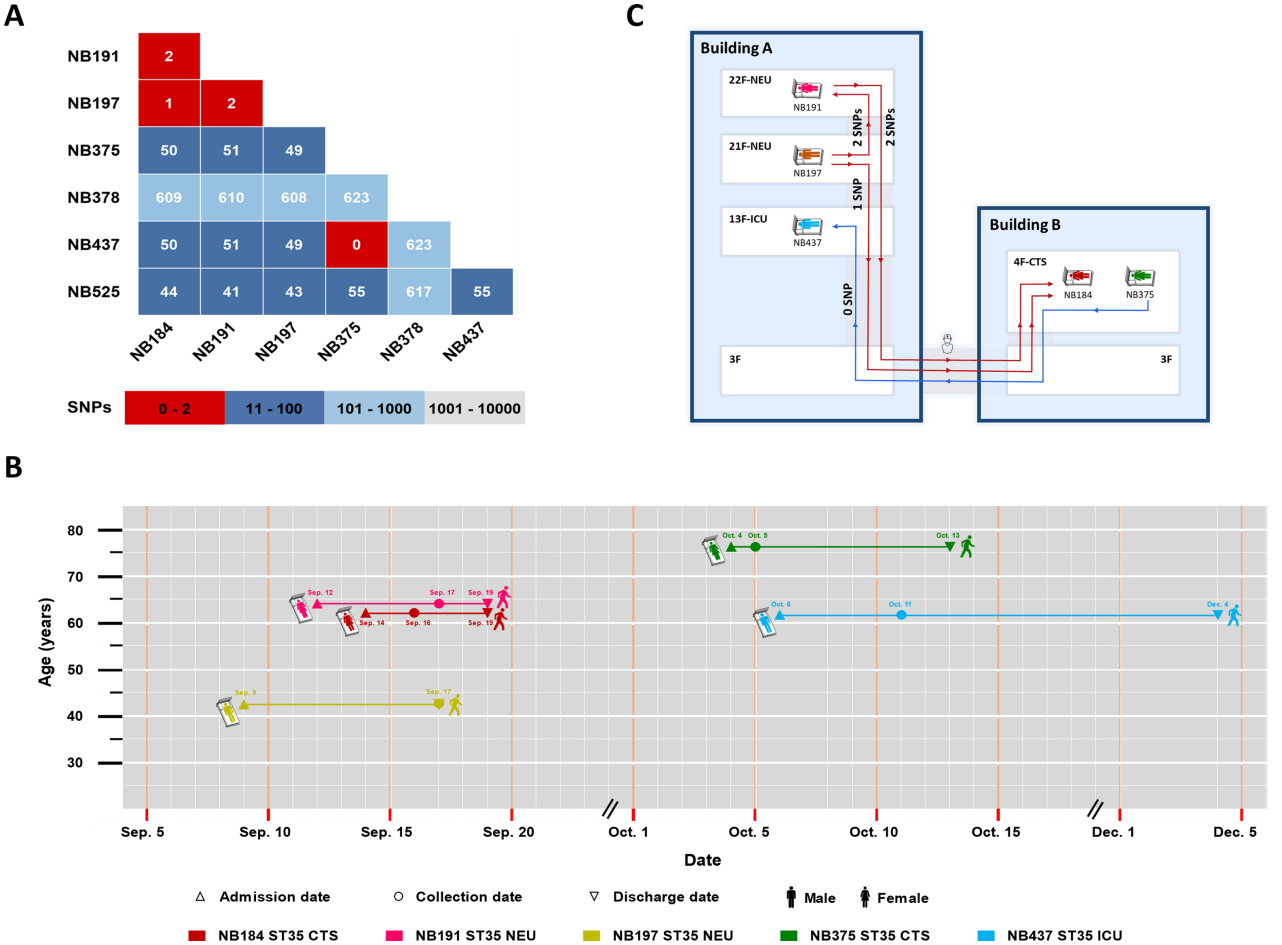
**(A)** The number of SNP differences in pairs was determined in the genomes of seven ST35 strains; **(B)** The timelines of five hospitalized patients with genetically related strains. Patient admission dates are marked by equilateral triangles, collection dates by circles and discharge dates by inverted triangles; **(C)** The transmission map of two nosocomial clonal transmission events among different floors and buildings. CTS, cardiothoracic surgery department; NEU, neurology department; ICU, intensive care unit.

WGS and SNP analysis was performed on 21 strains with identical STs from the same ward, including 7 strains of ST35, 6 of ST36, 4 of ST3, 2 of ST14, and 2 of ST54. These strains were collected within a 124-day period. The genome sequences of the seven ST35 strains were aligned to the *C. difficile* ST35 complete genome A9 (SRR18235872). Among these, three strains (NB184, NB191, and NB197) showed ≤2 SNP differences, while no SNP difference was found between NB375 and NB437 (Figure 4A). By integrating SNP results with patient hospitalization timelines (Figure 4B), two nosocomial clonal transmission events were identified (Figure 4C). The first event occurred between September 9 and 17, involving the spread of *C. difficile* ST35 between patient NB197 and NB191 in the NEU, and subsequently to NB184 in the CTS. The second event, occurring between October 4 and 11, involved the clonal transmission of *C. difficile* ST35 between patient NB437 in the CTS and NB375 in the ICU. In addition, two ST36 strains (NB454 and NB647), which were isolated less than 124 days apart, had 8 SNP differences. The genome sequences of the other non-ST35 strains were aligned to the *C. difficile* 630 genome (AM180355.1) (Supplementary Figure S2).

### 3.3 Risk factors

Risk factors for CDI were assessed through a blinded review of each patient’s medical records. Bivariate analysis was performed between the 115 CDI cases and 792 non-CDI cases, as shown in Table 2. The following parameters were found to be statistically significant between CDI cases and non-CDI cases: hospitalization over 10 days before sampling (OR = 1.757, 95% CI = 1.053–2.931, *p* = 0.029), the use of penicillin-class antibiotics (OR = 1.690, 95% CI = 1.107–2.579, *p* = 0.019), and use of esomeprazole (OR = 1.942, 95% CI = 1.159–3.253, *p* = 0.014). Subsequent bivariate logistic analysis showed that the use of penicillin-class antibiotics remained a significant factor associated with CDI (OR = 1.573, 95% CI = 1.020–2.424, *p* = 0.040).

**Table 2:** Parameters and risk factors in 907 hospitalized patients with diarrhea.

Parameter	CDI cases (*n* = 115)	Non-CDI cases (*n* = 792)	Analysis results					
			Bivariate			Bivariate		
			OR	95% CI	*p* value	OR	95% CI	*p* value
Age > 60 yrs. (median, 60 yrs.)	52 (54.8)	386 (51.3)	1.152	0.778–1.706	0.480			
Gender, male	74 (64.3)	464 (58.6)	1.276	0.849–1.917	0.264			
Previous antibiotic treatment within 8 week								
Cephalosporin class (third and fourth generations)	49 (42.6)	330 (41.7)	1.039	0.700–1.544	0.919			
Quinolone class	11 (9.6)	60 (7.6)	1.290	0.657–2.534	0.578			
Carbapenem class	18 (15.7)	88 (11.1)	1.485	0.857–2.572	0.163			
Penicillin class	38 (33.0)	179 (22.6)	1.690	1.107–2.579	0.019	1.573	1.020–2.424	0.040
Others	4 (3.5)	15 (1.9)	1.867	0.609–5.725	0.286			
Hospital stay >10 days before sampling, yes	22 (19.1)	94 (11.9)	1.757	1.053–2.931	0.029	1.533	0.907–2.594	0.111
Past medical history								
Tumor	36 (31.3)	189 (23.9)	1.454	0.949–2.228	0.105			
Infectious diseases	31 (27.0)	173 (21.8)	1.320	0.846–2.061	0.232			
Surgery	22 (19.1)	218 (27.5)	0.623	0.382–1.017	0.070			
Chronic disease	26 (22.6)	212 (26.8)	0.799	0.502–1.272	0.367			
Ward type								
GAS	26 (22.6)	146 (18.4)	1.293	0.806–2.073	0.308			
HEM	12 (10.4)	64 (8.1)	1.325	0.692–2.539	0.470			
HBS	10 (8.7)	63 (8.0)	1.102	0.548–2.214	0.854			
INF	9 (7.8)	39 (4.9)	1.639	0.772–3.480	0.261			
ICU	9 (7.8)	61 (7.7)	1.017	0.491–2.109	1.000			
CTS	8 (7.0)	75 (9.5)	0.715	0.335–1.523	0.399			
NEU	6 (5.2)	63 (8.0)	0.637	0.269–1.507	0.352			
Others	35 (30.4)	281 (35.5)	0.796	0.521–1.215	0.298			
Surgery during hospitalization, yes	38 (33.0)	300 (37.9)	0.809	0.535–1.225	0.353			
Nasal feeding, yes	11 (9.6)	69 (8.7)	1.108	0.568–2.163	0.860			
Previous PPIs use								
Pantoprazole	37 (32.2)	301 (38.0)	0.774	0.510–1.174	0.257			
Esomeprazole	22 (19.1)	86 (10.9)	1.942	1.159–3.253	0.014	1.699	0.995–2.902	0.052
Omeprazole	21 (18.3)	163 (20.6)	0.862	0.521–1.426	0.621			
Clinical laboratory tests								
WBC (cells ×10^9^/L) >10	20 (17.4)	116 (14.6)	1.227	0.729–2.065	0.484			
Neutrophils >75%	36 (31.3)	201 (25.4)	1.340	0.876–2.050	0.211			
ALT (U/L) > 50	12 (10.4)	84 (10.6)	0.982	0.518–1.861	1.000			
AST (U/L) > 40	14 (12.2)	96 (12.1)	1.005	0.552–1.828	1.000			
Albumin (g/L) < 40	48 (41.7)	289 (36.5)	1.247	0.838–1.856	0.302			
Urea (mmol/L) > 9.5	11 (9.6)	70 (8.8)	1.091	0.559–2.128	0.861			
Creatinine (μmol/L) > 111	10 (8.7)	74 (9.3)	0.924	0.463–1.845	0.866			
CK-MB (ng/ml) > 5	1 (0.9)	21 (2.7)	0.322	0.043–2.417	0.344			

Other antibiotics included glycopeptides, tetracyclines, and lincosamides; GAS, gastroenterology department; HEM, hematology department; HBS, hepatobiliary surgery department; INF, infectious diseases department; ICU, intensive care unit; CTS, cardiothoracic surgery department; NEU, neurology department; Other departments included obstetrics and gynecology, pediatrics, and geriatrics; PPI, Proton pump inhibitor; WBC, white blood cell; ALT, alanine aminotransferase; AST, aspartate aminotransferase; CK-MB, creatine kinase isoenzyme.

Significant differences were revealed between 15 ST35 and 100 non-ST35 CDI cases in rates of admission to the ICU (OR = 12.000, 95% CI = 2.767–52.047, *p* = 0.002) and the NEU (OR = 8.083, 95% CI = 1.463–44.648, *p* = 0.037). Subsequent bivariate logistic analysis showed that admission to the NEU was a significant risk factor between ST35 and non-ST35 CDI cases (OR = 1.556, 95% CI = 1.011–2.396, *p* = 0.044).

## 4 Discussion

The previous study did not address CDI risk factors among hospitalized patients in Ningbo, China (Shu et al., 2023). To fill this knowledge gap, we conducted a further analysis of molecular characteristics, clonal transmission, and risk factors in a tertiary hospital. Our study provided the first genomic evidence of *C. difficile* ST35 among different floors and buildings within the hospital. Notably, NEU admission was identified as an independent risk factor for *C. difficile* ST35 infection, informing prevention strategies for large-scale outbreaks in this region.

This study revealed a CDI prevalence of 12.7%, comparable to rates reported among Chinese patients with diarrhea in a meta-analysis (11.4%) (Wen et al., 2023). Our previous cross-sectional study (2013–2015) identified *C. difficile* ST37 as a dominant genotype (16.5%) (Jin et al., 2017), contrasting with the current study, which found ST37 at a prevalence of 1.7%. *C. difficile* ST81, a single-allelic *atpA* variant of ST37, has been predominant in Shanghai for a decade (Yang et al., 2020), but our study found only 0.9% of strains were ST81. A recent meta-analysis observed significant molecular differences in *C. difficile* between northern and southern China (Wen et al., 2023). Regional variations might be attributable to differences on genotypes and antibiotic use patterns in *C. difficile* epidemiology (Luo et al., 2019). Therefore, further regional studies are crucial to fully elucidate CDI molecular characteristics in this region and understand the factors driving these regional variations in China.

Our study revealed significant changes in antibiotic resistance patterns of *C. difficile* strains, highlighting the dynamic nature of CDI epidemiology in this region. The antibiotic resistance pattern in this study showed that the rates of resistance to ciprofloxacin, levofloxacin, and tetracycline were significantly lower than data from our previous study (Jin et al., 2017). While the rates on clindamycin resistance were prevalent in Asia-Pacific countries (80.7%) and China (70.8–87.9%), the quinolone resistance rates (24.3–34.8%) in this study were lower than those reported in China (Luo et al., 2019; Wu et al., 2022). Similarly, the tetracycline resistance rate (6.1%) aligned with the coastal region data but differed from other domestic studies (6.0–46.9%) (Yan et al., 2017; Wu et al., 2022). These findings highlighted variations in CDI epidemiology and antibiotic resistance patterns in this region. Notably, we identified one ST1 isolate with a metronidazole MIC value of 2 μg/mL. The emergence of metronidazole-resistant *C. difficile*, predominantly *C. difficile* ST1, has been a growing concern worldwide (Abdrabou et al., 2021). Given that metronidazole is currently not recommended as a first-line drug according to the IDSA/SHEA guideline in 2017 (McDonald et al., 2018), dynamic changes in its MIC values should be continuously monitored.

Our study unveiled novel nosocomial clonal transmission among different floors and buildings within the hospital of *C. difficile* ST35, highlighting the complex dynamics of its spread in healthcare settings. *C. difficile* ST35 has caused severe outbreaks with high mortality in Sweden (Magnusson et al., 2022), and evidence suggested its widespread proliferation in Zhejiang through nosocomial and cross-species transmission (Luo et al., 2024). We identified two nosocomial clonal transmission events within a three-month period. This finding aligned with previous observations of *C. difficile* ST37 transmission among different floors in NEU departments in Zhejiang (Bi et al., 2023). Our epidemiological evidence suggested that cross-care provided by healthcare workers in the NEU likely served as the primary vector for this clonal transmission. Notably, we identified clonal transmission between patients NB191 and NB197 in the NEU and patient NB184 in the CTS, demonstrating bacterial spread among different floors and buildings within the hospital. Laboratory samples from both buildings were transported by the same staff, facilitating potential transmission among buildings. These observations indicated that healthcare worker mobility, environmental contamination, and indirect patient contact were key factors contributing to this clonal transmission.

The second clonal transmission event originated from patient NB437 from the CTS. Despite a one-day hospital stay, the patient, with three-year Crohn’s disease history, multiple prior admissions, and long-term use of antibiotics and immunosuppressants, might have been infected by *C. difficile* ST35 from other healthcare facilities prior to this admission. Subsequently, patient NB437 transmitted ST35 to susceptible ICU patient NB375 through transmission among buildings, linked to the same healthcare worker who transported the laboratory samples. Notably, *C. difficile* ST35 exhibited strong spore formation, a characteristic that significantly enhanced its environmental persistence and facilitates rapid spread in healthcare setting (Luo et al., 2024). These findings highlighted the need for strict infection control among healthcare workers moving among hospital areas, including enhanced hand hygiene and frequent disinfection of high-touch surfaces. Screening and monitoring ST35 cases on admission, especially those with inflammatory bowel disease, are also crucial.

For CDI risk, our findings corroborated previous reports identifying prolonged hospitalization (>10 days) as a risk factor for CDI (Yang et al., 2020). Bivariate logistic regression analysis revealed penicillin-class antibiotics as an independent risk factor for CDI, consistent with a study conducted in Hong Kong (Guo et al., 2021). Notably, all CDI patients in our study had a history of piperacillin-tazobactam usage, underscoring the necessity for stringent control and monitoring of this antibiotic to mitigate CDI risk. Further analysis of all *C. difficile* ST35 cases revealed novel risk factors. ICU admission was associated with increased ST35 infection risk, possibly due to the compromised immune status of ICU patients and frequent use of broad-spectrum antibiotics (Murphy et al., 2022). Notably, admission to the NEU emerged as an independent risk factor for ST35 infection. Recent research has shown that neurons play a crucial role in regulating CDI-induced inflammation (Manion et al., 2023), indicating neurological dysfunction may influence host susceptibility to CDI. However, the exact mechanisms warrant further investigation. These findings underscored the importance of judicious piperacillin-tazobactam use, routine CDI screening in high-risk patients, especially in the NEU, and prompt implementation of infection control measures.

This study has some limitations. First, clinical samples were collected over a three-month period, potentially missing seasonal variations and long-term molecular trends. Second, the absence of environmental sampling limited the understanding of direct nosocomial transmission pathways for ST35 strains. Additionally, the lack of clinical outcome data for ST35 cases limited the assessment of this genotype’s impact on disease severity.

In conclusion, this study elucidated multiple *C. difficile* genotypes were prevalent with varied antibiotic resistance patterns in Ningbo, China, and provided the genomic evidence of *C. difficile* ST35 clonal transmission among different floors and buildings within the hospital. Our findings also highlighted the importance of enhanced surveillance and targeted interventions for penicillin-class antibiotics use, and *C. difficile* ST35 cases in the NEU. Further studies should be conducted to investigate long-term molecular trends, environmental reservoirs, and clinical outcomes to comprehensively understand CDI dynamics and inform prevention strategies.

## Data Availability

The datasets presented in this study can be found in online repositories. The names of the repository/repositories and accession number(s) can be found in the article/Supplementary material.

## Footnotes

^1^ http://pubmlst.org/cdifficile

^2^ www.eucast.org
