# Supplementary material for: Molecular characteristics, clonal transmission, and risk factors of Clostridioides difficile among hospitalized patients in a tertiary hospital in Ningbo, China
Source: Front Microbiol. 2024 Dec 2;15:1507128. doi: 10.3389/fmicb.2024.1507128 (PMC11648312; doi:10.3389/fmicb.2024.1507128)
Supplement: Supplementary file 1 [file Data_Sheet_1.docx]

Supplementary Material

# Supplementary Table

## Table S1 Clinical information of all 907 hospitalized patients

| **Patient characteristics** |  |
| --- | --- |
| **Demographics** |  |
| Age [mean, median (range)] (year) | 57.8, 60.0 (1-96) |
| Gender Male [*n* (%)] | 538 (59.3%) |
| **Age group [n (%)]** |  |
| <2 | 16 (1.8%) |
| 2-18 | 21 (2.3%) |
| 19-50 | 236 (26.0%) |
| 51-70 | 394 (43.4%) |
| >70 | 240 (26.5%) |
| **Department type [n (%)]** |  |
| GAS | 172 (19.0%) |
| CTS | 83 (9.2%) |
| HEM | 76 (8.4%) |
| HBS | 73 (8.0%) |
| ICU | 70 (7.7%) |
| NEU | 69 (7.6%) |
| INF | 48 (5.3%) |
| Others | 316 (34.8%) |
| **Underlying disease [n (%)]** |  |
| Tumor | 225 (24.8%) |
| Infection | 204 (22.5%) |
| Surgery | 240 (26.5%) |
| Chronic disease | 238 (26.2%) |
| **Notes:** GAS, gastroenterology department; HEM, hematology department; HBS, hepatobiliary surgery department; INF, infectious diseases department; ICU, intensive care unit; CTS, cardiothoracic surgery department; NEU, neurology department; Other departments included obstetrics and gynecology, pediatrics, and geriatrics. | |

## Table S2 Correlations between genotypes and antibioticsusceptibility patterns of *C. difficile* strains

| **Antibiotic agent***^a^* | **Total no. (%) of strains**  **(n = 115)** | **Genotype (no. [%] of nonsusceptible strains)** | | | | | **Analysis results***^b^* | |  |
| --- | --- | --- | --- | --- | --- | --- | --- | --- | --- |
|  |  | **ST2**  **(n = 12)** | **ST3**  **(n = 16)** | **ST35**  **(n = 15)** | **ST54**  **(n = 11)** | **Other STs (n = 61)** | **χ^2^** | ***P* value** |  |
| Metronidazole | 0 | 0 | 0 | 0 | 0 | 0 | — | — |  |
| Vancomycin | 0 | 0 | 0 | 0 | 0 | 0 | — | — |  |
| Clindamycin | 82 (71.3) | 7 (58.3) | 12 (75.0) | 10 (66.7) | 10 (90.9) | 43 (70.5) | 1.60 | 0.66 |  |
| Erythromycin | 63 (54.8) | 5 (41.7) | 10 (62.5) | 7 (46.7) | 11 (100.0) | 30 (49.2) | 10.24 | 0.02 |  |
| Fusidic acid | 52 (45.2) | 3 (25.0) | 6 (37.5) | 9 (60.0) | 5 (45.5) | 29 (47.5) | 3.58 | 0.31 |  |
| Rifampin | 5 (4.3) | 0 | 0 | 0 | 1 (9.1) | 4 (6.6) | 3.98 | 0.26 |  |
| Tetracycline | 7 (6.1) | 0 | 1 (6.3) | 5 (33.3) | 0 | 1 (1.6) | 20.19 | < 0.001 |  |
| Piperacillin | 1 (0.9) | 0 | 0 | 0 | 0 | 1 (1.6) | — | — |  |
| Levofloxacin | 34 (29.6) | 3 (25.0) | 7 (43.8) | 0 | 2 (18.2) | 22 (36.1) | 7.63 | 0.05 |  |
| Moxifloxacin | 29 (25.2) | 0 | 8 (50.0) | 0 | 2 (18.2) | 19 (31.1) | 16.65 | 0.001 |  |
| Gatifloxacin | 28 (24.3) | 0 | 7 (43.8) | 0 | 2 (18.2) | 19 (31.1) | 14.91 | 0.002 |  |
| Ciprofloxacin | 40 (34.8) | 4 (33.3) | 5 (31.3) | 10 (66.7) | 1 (9.1) | 20 (32.8) | 13.30 | 0.004 |  |
| MDR | 80 (69.6) | 5 (41.7) | 12 (75.0) | 9 (60.0) | 11 (100.0) | 43 (70.5) | 9.88 | 0.02 |  |
| *^a^* MDR: multidrug resistant  *^b^* —, data not calculated | | | | | | | | | |
|  | | | | | | | | | |

# Supplementary Figure

**
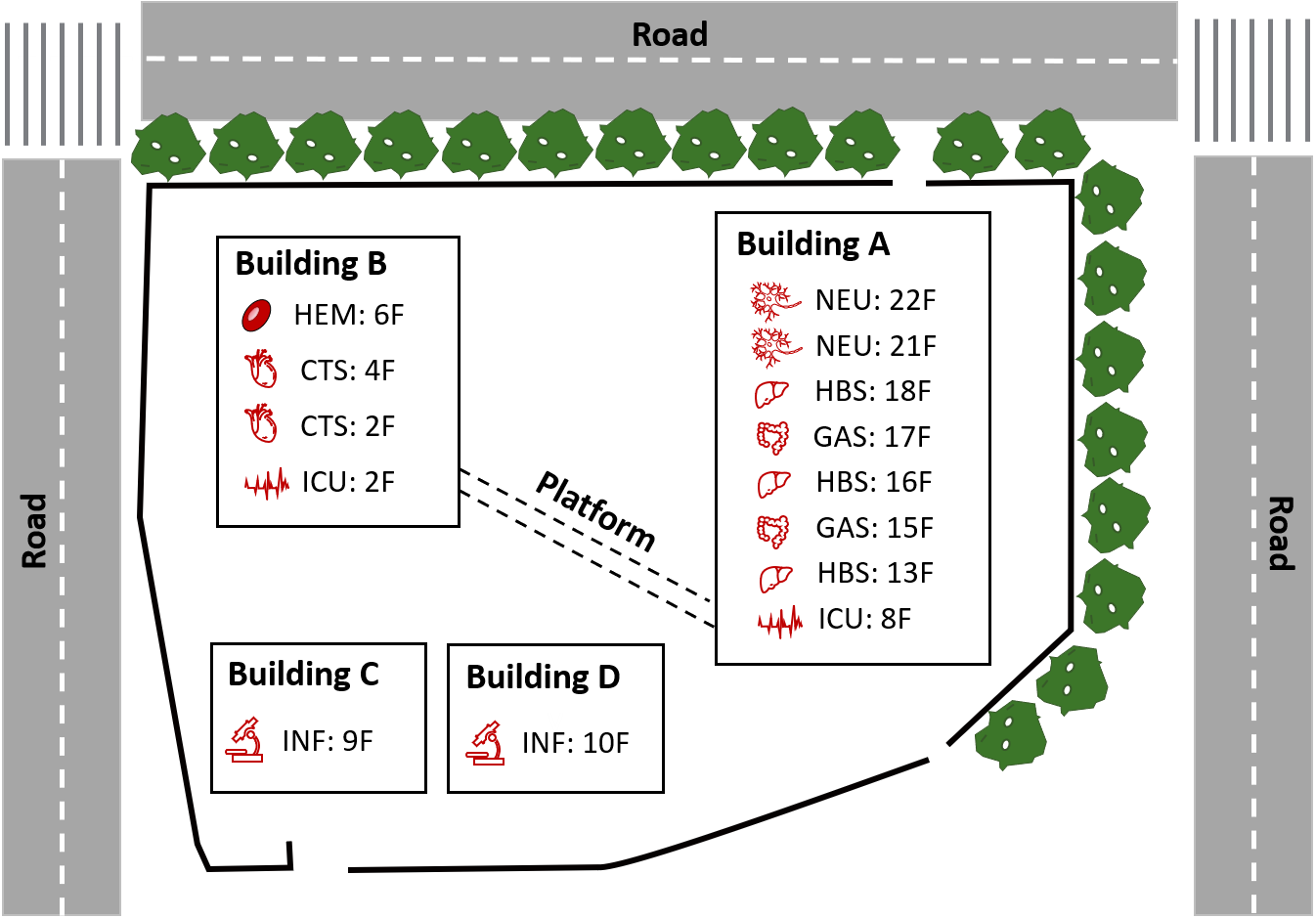
**

**Fig S1.** The distribution of the main hospitalized departments.

**
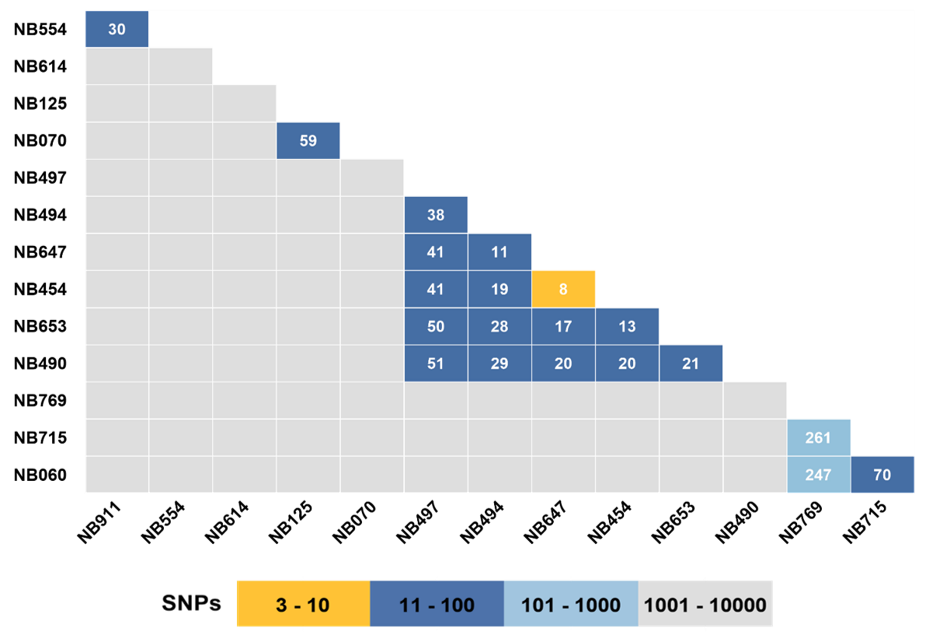
**

**Fig S2.** The SNP differences in non-ST35 isolates.
